# The Virtual Cell Animation Collection: Tools for Teaching Molecular and Cellular Biology

**DOI:** 10.1371/journal.pbio.1002118

**Published:** 2015-04-09

**Authors:** Katie M. Reindl, Alan R. White, Christina Johnson, Bradley Vender, Brian M. Slator, Phillip McClean

**Affiliations:** 1 Department of Biological Sciences, North Dakota State University, Fargo, North Dakota, United States of America; 2 Department of Biological Sciences, University of South Carolina, Columbia, South Carolina, United States of America; 3 Department of Plant Sciences, North Dakota State University, Fargo, North Dakota, United States of America; 4 Department of Computer Science, North Dakota State University, Fargo, North Dakota, United States of America

## Abstract

A cell is a minifactory in which structures and molecules are assembled, rearranged, disassembled, packaged, sorted, and transported. Because cellular structures and molecules are invisible to the human eye, students often have difficulty conceptualizing the dynamic nature of cells that function at multiple scales across time and space. To represent these dynamic cellular processes, the Virtual Cell Productions team at North Dakota State University develops freely available multimedia materials to support molecular and cellular biology learning inside and outside the high school and university classroom.

## Abstract 2

This piece from our Eduction Series describes the development of freely available multimedia materials to help molecular and cellular biology students conceptualize the dynamic nature of cells at multiple scales in time and space.

This Education piece is part of the Education series.

## The Value of Visualization

Molecular and cellular biology (MCB) processes occur at a scale and on a time frame impossible to see with the naked eye. With no direct visual reference, students rely upon visual representations to understand complex MCB processes. In a static image, students must be able to interpret the image in a meaningful way and imagine how the molecules interact in processes over time. In contrast, animations provide students with a dynamic model showing how interactions take place within their appropriate subcellular context. The utility of animations to supplement learning alongside static 2-D images is evident in that nearly all modern biology textbooks include a media CD or DVD or online access to animations. Furthermore, a large number of research studies have emphasized the importance of animations for the understanding of biology concepts [1], for their value for long-term memory retention [2], and as media able to engage students to a greater extent than textbook materials [3,4]. Particularly, animations appear to be superior to static images for learners with low spatial-visualization ability [5]. Collectively, the evidence convincingly shows that animations are useful tools for learning the complex biology content taught in high school and college classrooms.

## Development of the Virtual Cell Productions Collection

The Virtual Cell Productions team began animation development in 2002, using 3-D models originally designed to guide the development of an immersive role-playing educational game [6]. Those models and the vast collection developed later were critical to the development of dynamic education media. The Virtual Cell animations (Box 1) are designed to deliver comprehensive content related to core MCB topics appropriate for advanced high school or university introductory biology courses and are intended to help students learn the key structures and molecules of a MCB process and how these interact over time and space. For the most part, the animations illustrate whole processes (translation, mitosis, electron transport, etc.) rather than specific events or concepts within a process (conformational shape change of enzymes, microtubule assembly or disassembly, etc.). Because our animations provide a high level of scientific detail for each process, most students need to view the animations multiple times to fully understand and learn the content. These animations are available from the project site and a number of locations on the World Wide Web (Box 2).

**Box 1. Concepts at a Glance**

The Virtual Cell Animation Collection currently contains 24 MCB animations including nine cellular processes, six molecular processes, eight cellular energy conversion topics, and an introductory animation fly-through tour of a cell [8]. The collection is targeted primarily toward advanced high school and college undergraduate biology courses. The duration of the animations is shown in parentheses and ranges from about 2–7 min.

Introduction to a Cell

Through the Virtual Cell (6:45) α, β

Cellular Processes

Protein Trafficking (Golgi) (3:27) β, γ

Protein Modification (3:49) β, γ

Protein Recycling (3:15) γ

Insulin Signaling (4:42) β, γ

Constitutive Secretion (3:29) γ

Regulated Secretion (3:24) γ

Mitochondrial Protein Transport (3:22) γ

Mitosis (6:10) α, β, γ

Meiosis (5:27) α, β, γ

Molecular Processes

RNA Transcription (2:50) α, β, γ

Regulated Transcription (3:36) γ

mRNA Processing (2:30) β, γ

mRNA Splicing (2:55) β, γ

Protein Translation (3:32) α, β, γ

Bacterial Gene Expression/Lac Operon (3:23) α, β, γ

Cellular Energy Conversion

Biological Gradients/ATP Synthase (3:47) α, β, γ

Cellular Respiration/Electron Transport (3:49) α, β, γ

Photosynthesis (Light Reactions) (5:04) α, β, γ

Photosystem II (4:31) γ

Glycolysis (Overview) (3:10) α, β

Glycolysis (Reactions) (5:09) γ

Citric Acid Cycle (Overview) (3:17) α, β

Citric Acid Cycle (Reactions) (4:24) γ

α = for high school biology courses

β = for introductory college biology courses

γ = for advanced college biology courses

**Box 2. Teaching Tools**

Multimedia links: The Virtual Cell (VCell) multimedia contents are freely available through a number of different sources.

VCell home page http://vcell.ndsu.nodak.edu/animations/

Download site http://vcell.ndsu.nodak.edu/animations/downloads/

YouTube page http://www.youtube.com/user/ndsuvirtualcell

MERLOT http://www.merlot.org/merlot/viewMaterial.htm?id=82021

VCell App http://itunes.apple.com/us/app/virtual-cell-animations/id427893931

**Fig 1:**
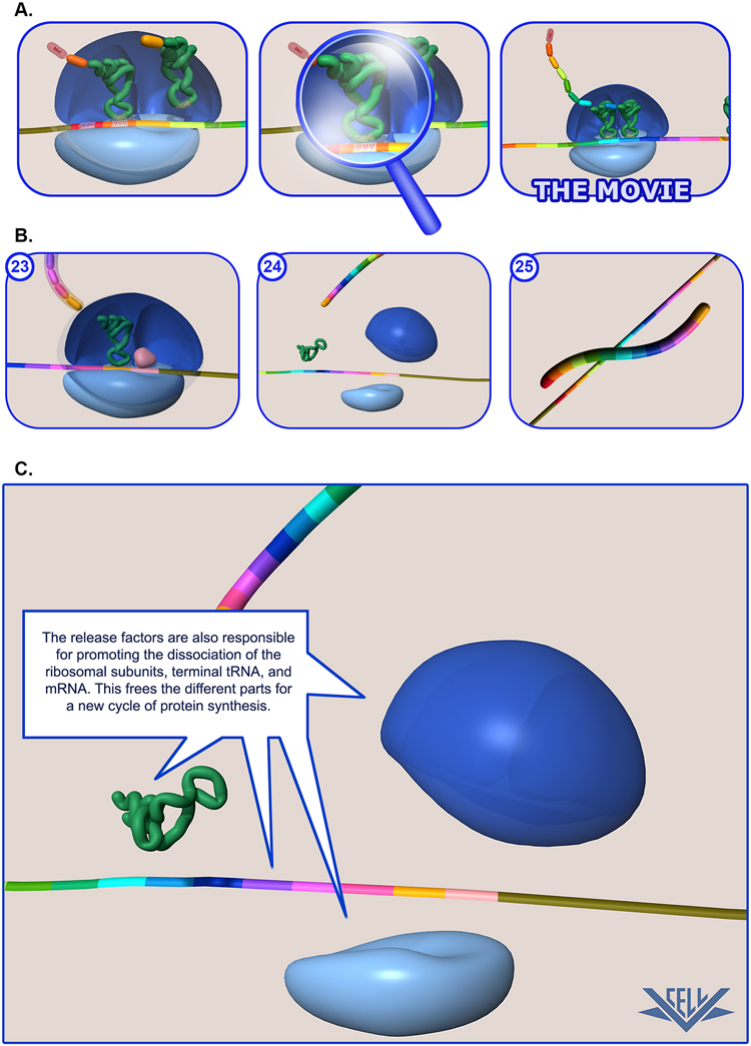
Education levels of Virtual Cell animations. (A) The Virtual Cell multimedia content is presented with two levels of supporting detail, First Look and Advanced Look, each tailored to student learning levels. The First Look provides a basic overview of the content appropriate for high school or first-year college students. The Advanced Look provides a more in-depth look at the content and additional details taught in advanced college courses and graduate and professional schools. The animations are offered in multiple formats (XviD AVI, Windows WMV, QuickTime MOV, and Flash FLV) to provide flexibility and can be downloaded and incorporated into various multimedia software in the case of a poor or slow internet connection. (B) Selection of the First Look or Advance Look brings up still images for a topic along with a brief description for the key events represented in each image. (C) Each still image is hyperlinked to a larger still image that includes labels for key features and further details about the topic. Image credit: North Dakota State University (NDSU) VCell Animation Project.

To supplement the animations, we extract still imagery from the animations with two levels of complexity so that the learner may view a key event without the added complexity of moving parts (Fig. 1). Students may also view more detailed annotated stills or read the animation narrative. Providing students with these choices enables greater flexibility and adaptability to various learning styles and preferences. All of these resources are also available from an individual page on the project web site (see Box 2).

Since their initial release, the animations in the Virtual Cell Collection have been subject to very specific design choices related primarily to the project’s target audience: introductory biology students at the late secondary and early collegiate levels. Most of these students have no previous or in-depth knowledge of the concepts being featured in the animations, and experience suggested many struggled to grasp the overall processes presented in their textbooks or lectures. The Virtual Cell animations aim to provide a stepping stone to more complicated and detailed explanations of individual components of a larger process—many of which can already be found elsewhere in both text and video formats (Howard Hughes Medical Institute [HHMI] BioInteractive, molecularmovies.com, YouTube, etc.). The Virtual Cell animations are geared toward helping novice learners quickly and successfully develop an introductory understanding of very detailed concepts. In most of the animations, the complexes, molecules, and their movements are illustrated in a highly stylized manner, with occasional nods to the known structures of proteins or cellular complexes. While some might suggest this is misleading, the project believes that by keeping the animations intentionally unrealistic and spare in appearance, students are more likely to believe that they are viewing a sequence of images designed to help them grasp the basic spatial or chronological flow of a pathway process rather than a precise reflection of reality. Once that foothold has been established, it is relatively easy to ask them to expand that understanding to include the idea that the process does not happen linearly or happens in many locations simultaneously. Excluding higher-level details or simplifying movements and reactions can be very useful when it comes to introducing biological concepts and in no way prevents building on those concepts with additional advanced materials as a student's biology education progresses. Though some students are certainly capable of absorbing highly detailed explanations and visualizations early on in their education, many students cannot.

**Fig 2:**
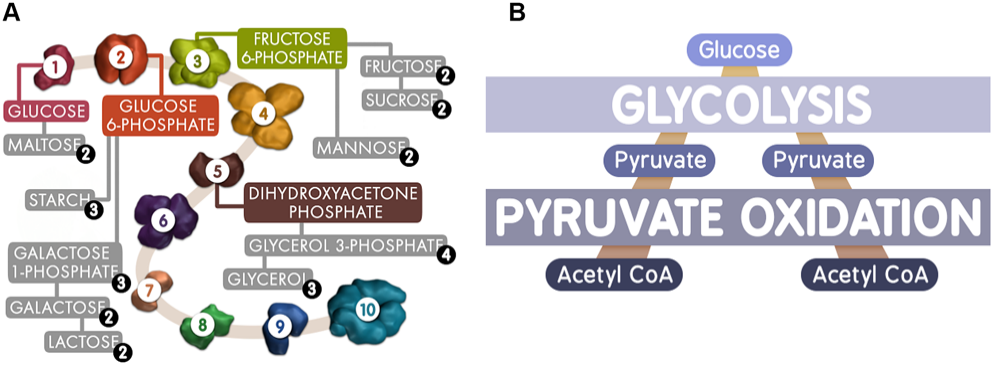
Multimedia strategy. Our recent development efforts have focused on incorporating real-world imagery and video into our animations. These visuals help the learner connect the invisible concepts of MCB with the world around them. (A) This image from the “Glycolysis: An Overview” animation details the substrate molecules for each step in the pathway (colored) and precursors (grey) of those substrates. (B) An additional developmental strategy has been the inclusion of infographics throughout our animations. In a manner that is appealing and memorable to modern students, this infographic rapidly communicates the basic fact that glucose flows through glycolysis and pyruvate oxidation. Image credit: NDSU VCell Animation Project.

The design principles for the animations were based on early educational research that identified features (audio narration, visual cues, labels, etc.) that make animations most effective for learning [7,8]. Mayer and Moreno’s theory of multimedia learning suggests that individuals learn best when (1) they are able to both see an animation and hear narration for that animation as opposed to animation and on-screen text, (2) they are pretrained by being presented with components of the system before seeing how all of the components work together in a process, (3) they are signaled by verbal or visual cues to focus in on important details, and (4) labels are positioned near their corresponding images [8]. These best practices are important for reducing cognitive overload that may occur with other animation styles that prevent the understanding of key concepts necessary for further learning [9]. Many MCB processes are interrelated and involve the same molecules, structures, or organelles. To visually emphasize these relationships, we developed a series of related animations with similar design features in which we recycle characters to create a collection of fluid MCB learning tools. To couple the animations with knowledge developed by structural biologists, the shapes of many of the molecular representations are based on 3-D structures found in the Protein Databank (PDB), a repository of more than 100,000 proteins and other biomolecules. Our static images are often similar to those used in textbooks and may help students make additional connections between animation content and content found in other learning tools. As mentioned above, the animations are targeted toward high school and first-year university-level biology learners. As such, the animations sometimes represent proteins or complexes in a simplified manner so that the learner is not distracted from developing a basic model of individual components and how those components work together in a system. Furthermore, our design approach has evolved to incorporate new design techniques used in modern media. Real-world imagery and infographics are two such trends. The “Mitosis” animation is an early example in which those techniques were incorporated. Fig. 2 highlights imagery extracted from the “Glycolysis: An Overview” animation and illustrates how we use infographics to help students connect a critical MCB pathway to the world around them.

**Fig 3:**
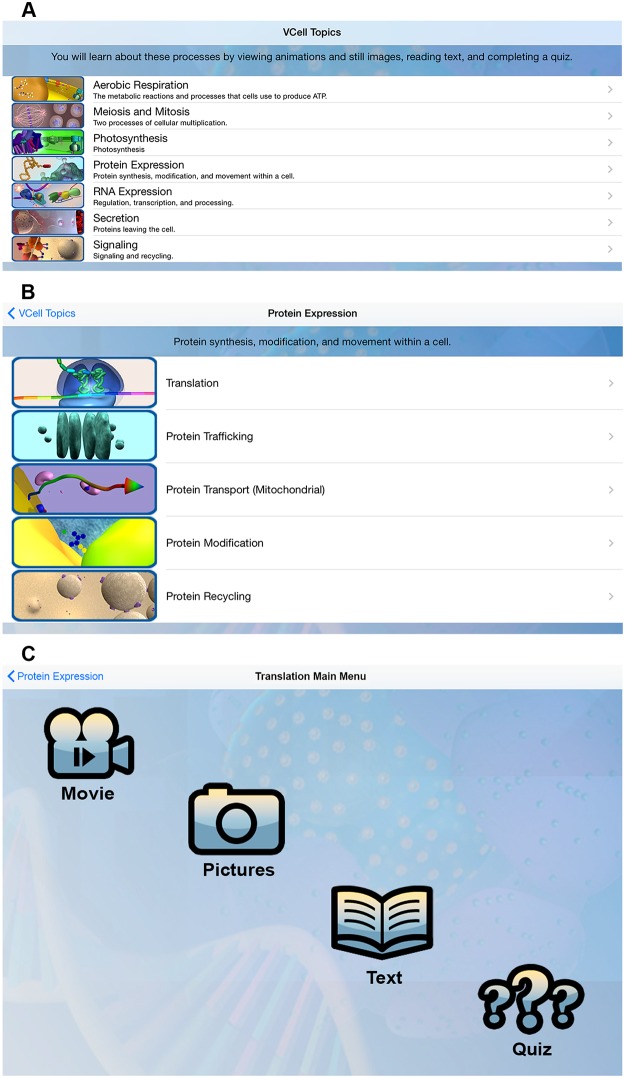
The Virtual Cell Animations application. The Virtual Cell Animation app is available for iOS devices as a mobile learning tool (https://itunes.apple.com/us/app/virtual-cell-animations/id427893931?mt=8). (A) The app contains the Virtual Cell multimedia collection assembled into seven different modules. These modules contain at least two related MCB topics to encourage students to think about the relationships between topics. (B) Here the Protein Expression module is shown below as an example. Five MCB processes related to protein expression, transport, modification, and activity are grouped together to help students understand how these topics are related to each other. The topics are organized in a chronological order of events; however, the student may view them in any order. (C) Once a topic within a module has been selected, the learner may choose to watch the animation, view still images, read the text associated with the animation, or take a short quiz to review the key concepts. The quiz provides feedback on selected responses and directs a user to animation clips or still images to reinforce topics. Image credit: NDSU VCell Animation Project.

Recognizing that learning can happen anywhere and at any time, we released the first version of the Virtual Cell application for devices using the iOS operating system in 2011 (Fig. 3). All of the topics available at the project web site were incorporated into the application. For each MCB topic, we have included the animation along with all of the learning materials available through our web site. As the user launches the application, they will find a series of higher-order MCB topics grouped into learning modules with the intent of highlighting how these topics are related to one another. The application also includes a self-guided quiz that provides feedback to student answers and directs them to the appropriate imagery and animation clips that address a quiz question.

## Best Uses of Animations for the Teacher and Learner

We are continually interested in how animations can best be used in traditional and contemporary course structures. A typical animation is about 4-min long (Box 1) and can be easily shown in the classroom as a preview to course content or to review material covered during the class period. Asking the students to answer basic questions during animation viewing or posing clicker questions following animation viewing helps students be more engaged while watching the animations during class. Outside of the classroom, the animations can be used prior to lecture for delivery of core content and to prime students for involvement in higher-order thinking activities within the classroom. The animations can also be used outside the class as a review of course material. Based on unsolicited feedback from our users, many students use the animations to help prepare for exams. For the majority of students, repeated viewing of the Virtual Cell animations is a requirement for successful learning. The learner likely needs to stop and start the animation multiple times as well as rewatch the animation in its entirety. Box 3 provides some ideas for how instructors can encourage their students to think critically while viewing animations inside or outside of class.

**Box 3. Learning Activities**

Activity 1: Learning key events of a process. Because the mechanics of a process are key to understanding a specific MCB topic, students can be encouraged to identify and discuss the following while viewing the animation:

- Key components involved in the process
- An overview of the process
- The order of major events
- The cellular location(s) where the events take place
- The relevance of the process for the development and/or reproduction of an individual

Activity 2: Identify threshold concepts and troublesome concepts.

Threshold concepts are perception gateways that transform the learner’s understanding or interpretation of a topic. Mastery of threshold concepts enables a learner to achieve higher-level views for that topic and helps a student understand troublesome concepts that are difficult to grasp. Animations, in combination with a guided learning activity (see Box 3 below), can be used to help students master threshold concepts to help students identify what concepts within a MCB process they find most challenging.

- Students complete a biology concept inventory pre-test.
- Instructor identifies troublesome concepts from the pre-test.
- Students are guided by the instructor through appropriate animations that feature the troublesome concepts.

Activity 3: Discover interrelations among processes.

To achieve a deeper understanding of an individual MCB process, students should be able to find parallels to other related processes.

- Students view several animations selected by the instructor.
- Instructor-guided discussion demonstrates to students how the topics are related.
- Example: Identify the similarities or differences between aerobic respiration and photosynthesis after viewing these animations.

## Research Results Using Virtual Cell Animations

The Virtual Cell Productions team has used our animation collection as research tools for a number of classroom experiments. Overall, our experiments indicate that (1) multiple viewing of animations increases learning gains beyond a single viewing before or during a lecture, (2) increased learning gains result from engagement with animations more than engagement with static graphics, and (3) students perceive that engagement with animations greatly facilitates their understanding of MCB processes ([1] and unpublished results).

Students learn less when they passively watch an animation [7]. We recognize the need for guided learning activities to supplement our animations. A guided activity can be used to walk a student through a topic as they are prompted to answer questions in response to reading text, viewing an animation, or interpreting data and figures. Several animations could be tied together to build a more comprehensive view of a larger process such as “cell signaling” (Box 4). During a guided activity, the animations help a student build a mental model for how a process works and enables them to use that model to understand how change to a system would affect a process.

**Box 4. Evaluation Tools**

Insulin signaling activity

To understand the complex topic of cell signaling, students must be able to connect several MCB processes together, including protein production, secretion, intracellular signaling, and resolution of the response. To aid student learning in a NDSU cell biology course, a guided learning activity was developed that included a series of questions that required students to analyze data, interpret images and graphs, and make predictions about the various processes involved in insulin signaling and the development of type I diabetes. Throughout the activity, students were encouraged, but not required, to view four Virtual Cell animations to help them build content knowledge regarding various aspects of insulin signaling. The four animations included “Protein Trafficking” (a protein is modified through the endomembrane system and packaged into secretory vesicles in the Golgi), “Regulated Secretion” (glucose regulates insulin secretion from a pancreatic cell), “Insulin Signaling” (insulin binds to its receptor on target cells and initiates a intracellular signal transduction pathway to mobilize glucose transporters to the cell surface), and “Protein Recycling” (glucose transporters are recycled back to a storage pool when glucose levels are low). By the end of the activity, students were asked to illustrate insulin signaling in a healthy individual compared to an individual with type I diabetes.

## Summary

The Virtual Cell multimedia collection provides freely available and cohesive tools for learning MCB processes, with an emphasis on the spatial and temporal relationships occurring during these processes, and is targeted toward high school and university-level introductory biology learners. Our development team has incorporated best practices for visualization design, including recent additions of real-world imagery, to maximize the impact on learning and to help students make connections between invisible MCB processes and the world around them [10]. The classroom experiments conducted using the Virtual Cell animations show positive effects on student learning [1]. Supplementing animations with guided learning activities may facilitate further educational gains.
